# Meningeal cryptococcosis and SARS-CoV-2 infection in people living with HIV/AIDS

**DOI:** 10.7705/biomedica.6872

**Published:** 2023-08-31

**Authors:** Fernando Antonio Messina, Andrés Benchetrit, Andrea Bocassi, María de las Mercedes Romero, Sofía Bauer, Emmanuel Marín, Facundo Bertera, Guillermo Onis, Matías Enzenhofer, Milagro Sánchez, Lilia Mammana, Dana Mijalovsky, Gabriela Santiso

**Affiliations:** 1 Unidad Micología, Hospital de Enfermedades Infecciosas Francisco Javier Muñiz, Buenos Aires, Argentina Hospital de Enfermedades Infecciosas Francisco Javier Muñiz Buenos Aires Argentina; 2 Sala 21, Hospital de Enfermedades Infecciosas F. J. Muñiz Hospital, Buenos Aires, Argentina Hospital de Enfermedades Infecciosas F. J. Muñiz Hospital Buenos Aires Argentina; 3 Laboratorio Central Hospital de Infecciosas, Francisco Javier Muñiz Hospital, Buenos Aires, Argentina Hospital de Infecciosas, Francisco Javier Muñiz Hospital Buenos Aires Argentina; 4 División SIDA, Hospital de infecciosas Francisco Javier Muñiz Hospital, Buenos Aires, Argentina Hospital de infecciosas Francisco Javier Muñiz Hospital Buenos Aires Argentina; 5 División Farmacia, Hospital de infecciosas Francisco Javier Muñiz Hospital, Buenos Aires, Argentina Hospital de infecciosas Francisco Javier Muñiz Hospital Buenos Aires Argentina; 6 Sala 20, Hospital de Infecciosas Francisco Javier Muñiz Hospital, Buenos Aires, Argentina Hospital de Infecciosas Francisco Javier Muñiz Hospital Buenos Aires Argentina; 7 Unidad Virología, Hospital de Infecciosas F. J. Muñiz Hospital, Buenos Aires, Argentina Hospital de Infecciosas F. J. Muñiz Hospital Buenos Aires Argentina

**Keywords:** COVID-19, SARS-Cov-2, Cryptococcus, meningitis, cryptococcal, coinfection, HIV infections, COVID-19, SARS Cov-2, Cryptococcus, meningitis criptocócica, coinfección, infecciones por VIH

## Abstract

**Introduction.:**

Fungal infections in patients with COVID-19 was one of the most debated topics during the pandemic.

**Objectives.:**

To analyze the clinical characteristics and evolution of people living with HIV/ AIDS and coinfection with cryptococcus and COVID-19 (group A) or without it (group B).

**Materials and methods.:**

This is an analytical and retrospective study. We reviewed medical records of patients with meningeal cryptococcosis between April 2020 and May 2021.

**Results.:**

We studied 65 people living with HIV/AIDS and with cryptococcosis infection diagnosed from April 2020 to May 2021. Fifteen patients with HIV/AIDS suffered from cryptococcosis and COVID-19, and out of these, 14 presented meningitis (group A), while 28 suffered from meningeal cryptococcosis, but did not have COVID-19 (group B).

**Conclusions.:**

No statistically significant differences were observed between the two groups (A and B) considering: intracranial hypertension, presence of *Cryptococcus* antigens in cerebrospinal fluid, sensorium deterioration or mortality. The detection of *Cryptococcus* antigens in serum by lateral flow assay was highly effective to rapidly diagnose cryptococcosis in patients with HIV/AIDS who also developed COVID-19. Patients of both groups consulted for cryptoccocosis sometime after, in comparison with the pre-pandemic cases related to this infection.

## Resumen

**Introducción.:**

Las infecciones fúngicas en pacientes con COVID-19 fue uno de los temas más debatidos durante la pandemia.

**Objetivo.:**

Analizar las características clínicas y la evolución de personas con VIH/SIDA que presentaron la asociación de criptococosis meníngea y COVID-19 (grupo A), y compararlas con aquellas personas con VIH/SIDA que padecieron criptococosis meníngea, pero sin infección de COVID-19 (grupo B).

**Materiales y métodos.:**

Se realizó un estudio analítico y retrospectivo en el que se revisaron las historias clínicas de pacientes que padecieron criptococosis meníngea entre abril de 2020 y mayo de 2021.

**Resultados.:**

Se estudiaron 65 pacientes con HIV/SIDA y con criptococosis, diagnosticados entre abril de 2020 y mayo de 2021 (63 habían desarrollado sida y 2 eran negativos para VIH). De estos, 15 de los pacientes con sida padecían criptococosis y COVID-19, y 14 presentaban meningitis (grupo A), mientras que 28 pacientes padecieron criptococosis meníngea, pero no tuvieron COVID-19 (grupo B).

**Conclusiones.:**

No se observaron diferencias estadísticamente significativas, entre los dos grupos, respecto a la hipertensión intracraneal, la presencia de antígenos de criptoccoco en líquido cefalorraquídeo, el deterioro del sensorio o la mortalidad. La detección de antígenos de *Cryptococcus* en suero por ensayo de flujo lateral fue efectiva para diagnosticar rápidamente criptococosis en personas con VIH/sida y con infección de COVID-19. Se observó que los pacientes de ambos grupos consultaron tarde por criptococosis en comparación con los casos prepandémicos de esta infección.

In December 2019, a new coronavirus, unknown until then and named SARS-CoV-2 was identified for the first time in Wuhan, China. Later, this virus caused an epidemic of an atypical pneumonia called Coronavirus Disease 2019 (COVID-19) ^1^^,^^2^. Due to its expansion speed and severity, on March 11, 2020, the World Health Organization (WHO) declared the pandemic ^3^.

The start of the pandemic raised the question about the evolution of SARS-CoV-2 infection in people living with HIV/AIDS, mainly those cases without antiretroviral treatment and low count of CD4+ lymphocytes. In this regard, these patients were initially considered at risk of suffering from severe clinical forms ^4^^,^^5^. With the course of the pandemic, different results on this subject were largely contradictory ^6^.

The association between fungal infections and COVID-19 has been the subject of multiple publications, especially for cases of aspergillosis, candidiasis, and mucormycosis ^7^^-^^9^. However, although the association between COVID-19 and cryptococcosis was reported in HIV-negative patients treated with immunomodulators ^10^ and in patients with autoimmune diseases ^11^, the number of cryptococcosis infections in people living with HIV/AIDS was low and, in general, they were excluded of the most recent reviews ^12^.

On the other hand, meningeal cryptococcosis in patients with HIV/AIDS is still one of the most frequent and serious opportunistic diseases in South America ^13^^,^^14^ as well as in the rest of the world ^15^.

The aim of this study was to compare the clinical characteristics and evolution of people living with HIV/AIDS and meningeal cryptococcosis in the presence (group A) or absence (group B) of COVID-19 infection.

## Materials and methods

This is an analytical and retrospective study. We reviewed the medical records of 65 patients with HIV/AIDS and cryptococcosis infection diagnosed between April 2020 and May 2021 in the Mycology Unit of our hospital.

### Inclusion criteria:

- People living with HIV/AIDS with meningeal cryptococcosis.

### Exclusion criteria:

- HIV-negative patients with cryptococcosis.
- People living with HIV/AIDS with extra-meningeal cryptococcosis.

We recorded data on age, sex, signs and symptoms, comorbidities, antiretroviral treatment, a physicochemical study of cerebrospinal fluid, opening pressure, antigenic score, lymphocyte populations count, and risk factors for HIV infection.

**Figure 1:**
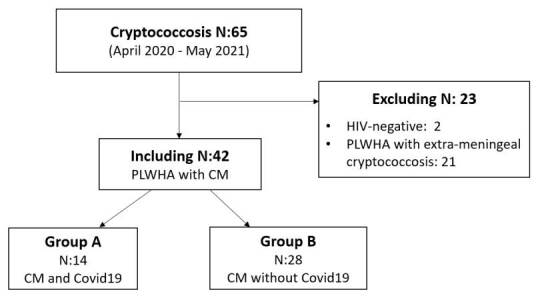
Flow chart with all the cryptococcosis patients (April 2020 - May 2021)

PLWHA: people living with HIV/AIDS; CM: meningeal cryptococcosis

The HIV-positive patients who met the inclusion criteria were divided into two groups. Group A included the patients who suffered from COVID-19, and group B, the patients who did not (figure 1).

### Mycological evaluation

Blood culture by lysis centrifugation was performed with samples from every patient with HIV/AIDS. The samples were cultured in Sabouraud-honey agar medium at 28°C and brain-heart infusion agar at 37°C for three weeks. Cultures were checked twice a week ^16^^,^^17^. In addition, *Cryptococcus* antigen detection in serum was performed by lateral flow assay (IMMY, Norman Kew Surrey, OK, USA) in patients with a CD4+ lymphocyte count down to 200 cells/μl ^18^^-^^20^.

For mycological examination, we took respiratory samples -sputum or bronchoalveolar lavage- from patients with respiratory symptoms or compatible images with pulmonary cryptococcosis. Likewise, we took a cerebrospinal fluid sample from patients with initial suspicion of central nervous system involvement.

All patients with a positive result for *Cryptococcus* antigen detection, cryptococcemia or pulmonary cryptococcosis underwent lumbar puncture to rule out or confirm meningeal involvement through mycological examination of cerebrospinal fluid.

All cerebrospinal fluid samples were microscopically tested with Indian ink and cultured on Sabouraud-honey agar, sunflower seed agar, and brain-heart infusion agar. Cultures were incubated at 28°C and 37°C for 15 days and were observed daily, seeking for colonies compatible with *Cryptococcus* spp.

Mycological control of patients with meningitis was performed with cerebrospinal fluid cultures at 2-3 weeks after the diagnosis and was repeated until a negative result was obtained.

### Identification tests

The phenotyping of the isolates was carried out by studying their growth capacity at 37°C, the presence of capsule, urease activity and phenoloxidase production in sunflower seeds agar. Differentiation between *Cryptococcus neoformans and Cryptococcus gattii* was performed by their growth in canavanine-glycine-bromothymol blue and Salkin’s medium (cycloheximideglycine-phenol red) respectively ^16^. Typification was confirmed by mass spectrometry (Vitek® MS). Strains were also genotypically studied by the amplification of the URA5 gene by PCR and subsequent restriction fragment length polymorphisms with the enzymes Sau96l and Hhal.

### Semi-quantitative detection of Cryptococcus capsular polysaccharide antigen

Blood samples were taken from all patients at the time of diagnosis. Serum was separated and kept at -20°C until the time of determination.

The semiquantitative determination of *Cryptococcus* capsular polysaccharide antigen in both serum and cerebrospinal fluid was carried out using the latex agglutination technique (Crypto Latex ®, IMMY, Norman Kew Surrey, OK, USA) according to the manufacturer’s instructions. Undiluted serum and 1:10; 1:100; 1:1000; 1:5,000 and 1:10,000 dilutions were used to determine the titer.

### Coinfection of Cryptococcosis meningitis and COVID-19

COVID-19 coinfection was confirmed in patients who presented a positive qRT-PCR for SARS-CoV-2 from a nasopharyngeal swab within 30 days of meningeal cryptococcosis having been diagnosed. We also included patients who persisted with development of *Cryptococcus*, from cerebrospinal fluid, in culture and COVID-19 diagnosed by qRT-PCR (positive for SARS-CoV2).

### Laboratory parameters

Erythrocyte sedimentation rate was evaluated on the device VES Matic Cube 300 (Diesse); D-dimer was determined by an enzyme-linked fluorescence assay using a VIDAS autoanalyzer; C-reactive protein, ferritin, and lactic dehydrogenase enzyme were analyzed on the Cobas C311 analytical platform by immunoturbidimetry and UV kinetic method for lactic dehydrogenase enzyme.

The leukocyte count was performed in the Sysmex XN-1000 hematology counter (optical/fluorescence impedance method). This can be used as an immune response severity index. The number of neutrophils, lymphocytes and the ratio between the two of them are markers of systemic inflammation.

### Severity criteria in meningeal cryptococcosis

In both groups we evaluated as severity criteria the following: intracranial hypertension, *Cryptococcus* antigen in cerebrospinal fluid greater than or equal to 1/5000, impaired sensorium and CD4+ lymphocyte count lower than 100 cells/mm^3^.

### Statistical analysis

Continuous variables were expressed as mean or median. Depending on the data distribution, we applied Student’s ttest or the Mann-Whitney- Wilcoxon to evaluate the statistical differences between groups A and B. Categorical variables were expressed as percentages and Fisher’s exact test was calculated. Differences were considered statistically significant when the pvalue was inferior to 0.05. We used Statistix® 8.0 software for analysis.

### Ethical considerations

We conducted this study according to the protocol and good clinical practice standards established by the research ethics committee of the Hospital Francisco Javier Muñiz.

## Results

A total of 15 patients with HIV/AIDS suffered from cryptococcosis and COVID-19. Fourteen had meningitis, and only one had positive antigenemia, found by lateral flow assay, with no other findings. Regarding gender, nine patients were men and six were women. The median age was 26.5 (range: 23-60).

None of the patients showed good adherence to antiretroviral therapy. Two of them were aware of their immunocompromised state at the time of hospitalization, and the remaining 12 were aware of their disease but were not under antiretroviral therapy.

**Figure 2:**
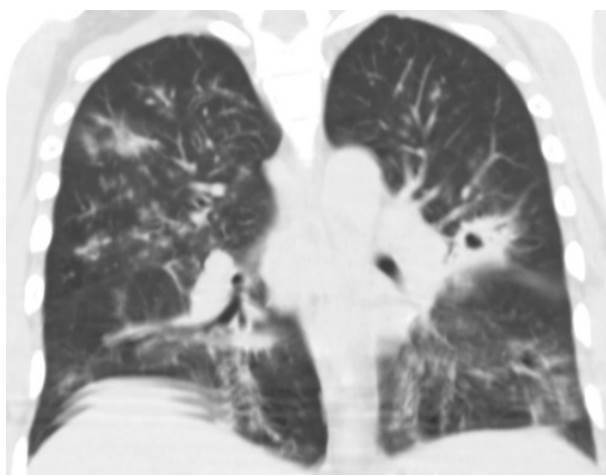
Chest computed tomography, without contrast enhancement, with ground glass lesions (compatible with COVID-19) and a cavitating nodule (compatible with pulmonary cryptococcosis).

Four patients presented pulmonary compromise compatible with COVID19 (figure 2). Out of these, three required mechanical ventilation and died. The brain tomography for the 14 patients with HIV/AIDS and meningitis showed no signs of lesions in the parenchyma. Also, none of them presented comorbidities related to severe COVID-19 (obesity, hypertension, heart failure, or diabetes). However, 13 of them had less than 100 CD4+ lymphocytes/μl.

Four patients had positive blood cultures for *Cryptococcus neoformans*. However, the pulmonary commitment by this microorganism was not confirmed in any patient, although we observed findings congruent with cryptococcosis in computed tomographies from three of them.

The three patients who died had positive cultures for *C. neoformans*, from cerebrospinal fluid samples, at the time of death. Nevertheless, it was impossible to establish whether they died from COVID-19 and/or cryptococcosis.

All isolates corresponded to the variety of *C. neoformans* VNI, according to phenotypic and genotypic identification.

Increased lactate dehydrogenase, dimer D and lymphopenia were the most frequent laboratory parameters in patients with COVID-19 and cryptococcosis (table 1). In patients without COVID-19, the lymphocyte count was not registered. Clinical and microbiological parameters related to severe meningeal cryptococcosis were similar in both groups. More patients with dyspnea were observed in the group with COVID-19 and cryptococcosis (group A) (table 2), probably because the lung is the main organ involved in patients with COVID-19, but this finding did not impact in the mortality rate of both groups.

**Table 1:** Laboratory parameters considered for severe COVID-19 and prevalence found in group A

Group A	n=14
Neutropenia	1/14
Lymphopenia	12/14
NLR^5.9	7/14
LDH>280 U/L	11/14
LDH>600 U/L	2/14
Ferritin>400 ng/ml	5/8
D-dimer>500 ng/ml	8/9
C-reactive protein>5 mg/L	4/5
ERS>25 mm/h	10/12

NLR: Neutrophil to lymphocyte ratio;

LDH: Lactic dehydrogenase; ERS:

Erythrocyte sedimentation rate

The main signs and symptoms of patients with meningeal cryptococcosis and COVID-19 in both groups are shown in table 2.

**Table 2:** Clinical and microbiological characteristics of the patients with meningeal cryptococcosis (group A, patients with COVID-19, and group B, patients without COVID-19)

	Group A n=14	Group B n=28	P value
Fever	13	24	0.2775
Headache	12	24	0.6471
Vomiting	11	17	0.1595
Dyspnea	7	2	0.0009*
Sensory impairment	5	8	0.54
Cough	3	2	0.1507
Elevated intracranial pressure	10	18	0.4629
CSF CrAg - LA >1/5000	8	17	0.9763
CD4+ < 100 cells/mm3	13	25	0.3912
Deceased	3	6	0.9129

CSF: cerebrospinal fluid; CrAg: cryptococcal capsular polysaccharide antigen; LA: latex agglutination test

* p<0.05

## Discussion

SARS-CoV-2 infection can cause asymptomatic clinical pictures as well as highly variable presentations. From myalgia, arthralgia, and anosmia to severe bilateral pneumonia ^21^. As it occurs in cryptococcosis, fever and headache were the most frequently observed symptoms in patients with HIV/AIDS with COVID-19 ^9^. For this reason, in the pandemic context, many patients attributed this symptomatology to COVID-19, but could have overlooked a possible fungal infection.

**Figure 3:**
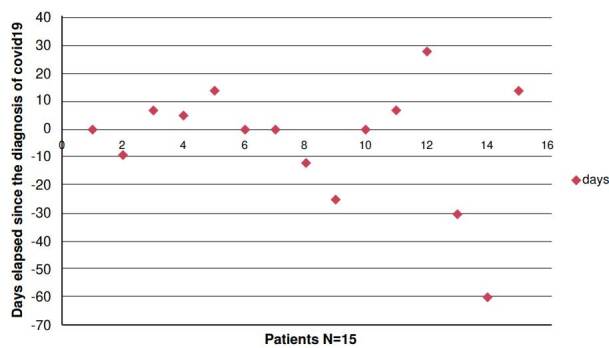
Relationship between the diagnostic time of cryptococcosis and COVID-19

For some years, *Cryptococcus antigen* detection, by lateral flow assay, has been routinely performed in patients with a CD4+ lymphocyte count less than or equal to 200 cells/μl, before starting antiretroviral treatment to prevent immune reconstitution syndrome ^20^. In the same way, it has been used in patients with a low CD4+ lymphocyte count when admitted with suspected opportunistic infection ^13^^,^^15^^,^^18^. This test provides a rapid diagnosis and the possibility to detect meningitis that otherwise, due to its scarce symptomatology focused on the central nervous system, would have a later detection. Figure 3 shows that most patients had a diagnosis of *Cryptococcus* meningitis close to the time when the nasopharyngeal admission swab was performed. Only few cases of COVID-19 infection occurred during hospitalization (figure 3). One patient was diagnosed with meningeal cryptococcosis after three weeks, because he was referred from a less complex center that did not have the *Cryptococcus* antigen detection test available.

Other cases undergoing meningeal symptoms and positive culture from cerebrospinal fluid, presented coinfection due to a nosocomial outbreak of COVID-19.

We detected an increase in the percentage of heavily treatmentexperienced patients (28/42 - 66 %) and presence of *Cryptococcus* antigens in cerebrospinal fluid greater or equal to 1/5000 (25/42 - 59.5 %), in both groups, compared with pre-pandemic data (heavily treatmentexperienced patients: 22/62 - 35.5 %; presence of *Cryptococcus* antigens ≥ 5000: 23/127 18.1 %) ^22^. This could be due to late consultation. However, considering the pre-pandemic meningeal cryptococcosis mortality (20.31 %) and the one observed during the pandemic (21.4 %), no significant differences were found ^22^.

Numerous authors support that the hyperinflammatory immune response induced by the SARS-CoV-2 virus plays a central role in the pathogenesis of the disease. In this work, we analyze the prognostic value of certain laboratory biomarkers in hospitalized patients with COVID-19. The recommendations suggest evaluating inflammatory markers such as: leukocyte differential count, erythrocyte sedimentation rate, D-dimer, ferritin, C-reactive protein, interleukin6, lactate dehydrogenase, procalcitonin, troponins and natriuretic peptides ^23^.

Most severe cases of COVID-19 presented a low absolute count of lymphocytes (lymphopenia <1000/mm^3^), increased leukocytes, a high absolute count of neutrophils, and increased neutrophil-to-lymphocyte ratio. It is important to mention that in this retrospective study interleukin6, procalcitonin, and cardiac biomarkers (troponins and natriuretic peptides) were not analyzed.

On the other hand, lymphopenia in patients with COVID-19 was associated with poor prognosis in different publications ^24^.

Throughout the pandemic, cut-off values for neutrophil-to-lymphocyte ratio between 3.0 and 6.0 have been reported. However, Sayah *et al*. have proposed neutrophil-to-lymphocyte ratio values greater than o equal to 5.9 to assess severity; and greater than or equal to 7.4 for mortality ^25^.

We observed lymphopenia in most patients but did not seem decisive in the outcome, probably because the number of patients studied was too small to reach conclusions. Furthermore, we clarify that we could not obtain the lymphocyte counts in patients with meningeal cryptococcosis but without COVID19 infection, a deficiency of this publication.

A recent meta-analysis of patients with COVID-19 shows a similar evolution of people living with HIV/AIDS with antiretroviral treatment compared with those living with HIV/AIDS but without antiretroviral therapy ^9^. In this study, despite their degree of immunocompromise, the evolution of patients living with HIV/AIDS was not worse than that of HIV-negative patients. However, the evidence regarding the relationship between people with HIV/AIDS and the increased risk of contracting or presenting severe forms of COVID-19 is still controversial. A recent study reported that those cases of COVID-19 with unfavorable evolution were related to comorbidities related to HIV.

A recent review on COVID-19 and cryptococcosis suggests that corticosteroid treatments may be a risk factor for developing this fungal disease. Unlike our cases, most patients in the mentioned review were negative-HIV patients with cardiovascular and metabolic comorbidities, suffering from pulmonary cryptococcosis ^26^.

The study of large prospective cohorts is necessary to differentiate the cryptococcosis impacts in HIVpositive and HIV-negative patients and thus develop some definitive recommendations.

Considering that predominant symptoms of cryptococcosis and COVID19 may overlap, the detection of *Cryptococcus* antigen by lateral flow assay from serum was relevant for a rapid diagnose in our patients.

The pandemic postponed consultation of most patients living with HIV/AIDS. Despite this, mortality in this period was similar to the one observed pre-pandemic.

No differences were observed between the mortality rate of patients with meningeal cryptococcosis and COVID-19 (group A) or without coinfection with COVID-19 (group B).
